# Early maladaptive schemas in overweight and obesity: A schema mode model

**DOI:** 10.1016/j.heliyon.2019.e02361

**Published:** 2019-09-17

**Authors:** Barbara Basile, Katia Tenore, Francesco Mancini

**Affiliations:** a Association of Cognitive Psychology (APC), School of Cognitive Psychotherapy (SPC), Rome, Italy; b Marconi University, Rome, Italy

**Keywords:** Eating disorders, Obesity, Early maladaptive schemas, Bingeing, Schema-modes, Clinical psychology, Psychiatry

## Abstract

Obesity is a growing burden in our societies and, although different kinds of treatments are effective in the short time, weight gain often reoccurs in the longer period. One possible explanation might rely on the little comprehension of obese maladaptive schemas, as developed from early life experiences, which might interfere with treatment enduring efficacy. The aim of this study was to investigate early maladaptive schemas, their associated current schema-modes and dysfunctional coping strategies in overweight and obese individuals (N = 48). Results showed that overweight and obese subjects reported more severe insufficient self-control, abandonment, dependence and subjugation schemas, and actual schema-modes (i.e., impulsive and vulnerable child, detached protector), compared against normal-weight controls (N = 37). As well, the former displayed higher dysfunctional eating habits (i.e., bingeing and bulimic symptoms) and more emotional-avoidant coping strategies. Above all schemas, insufficient self-control predicted higher BMI, binge frequency and bulimic symptoms' severity. Furthermore, avoidant coping mediated between specific maladaptive schemas and frequency of bingeing and bulimic symptoms. Our findings illustrate that overweight and obese display more dysfunctional early maladaptive schemas and schema-modes, compared against normal-weight individuals, exhibiting more emotion-avoidant strategies such as over-eating and bingeing, which might stand for a detached self-soother coping mode. The insufficient self-control schema develops from a lack in self-discipline and an inability to tolerate frustration and might be embodied by the impulsive child mode. A deeper comprehension of schemas and modes, as addressed within the Schema Therapy model, might help to understand dysfunctional personality features that might interfere with the long-lasting efficacy of treatment interventions in obesity.

## 1 Introduction

Obesity and overweight represent a growing burden in Western countries and while their biological and environmental factors are clearer and well known, few attention has been given to their psychological determinants, specifically considering deeper aspects of personality, rooted in early life experiences, that might predispose towards the chronicity of obesity. The most frequent treatments for obesity involve surgical and psychological interventions. The former include gastric bypass, sleeve gastrectomy, gastric banding and biliopancreatic bypass, while the latter refers to guided self-help [1], behavioral therapy [2] and cognitive-behavior therapy (CBT, [3, 4]). Both kind of interventions are quite efficient as most people who seek treatment lose weight in a short time, but unfortunately regain it shortly after [5, 6, 7, 8, 9]. One possible explanation of the lack of long-term outcomes might rely on the interference, within treatment, of early maladaptive schemas having developed in early life experience [10, 11]. These data suggest that dysfunctional eating patterns and habits associated with overweight and obesity are more deeply rooted within patients’ personality features, and that an *ad hoc* intervention might not *per s*e be enough to guarantee a long lasting effect. Hence, a deeper understanding of the emotional and psychological functioning of obese patients, also considering the role of early life experiences, might be helpful to address personality features and routed eating habits.

Schema Therapy (ST, [11]) is an integrated cognitive-behavioral and emotion-focused model that links actual psychological features and problems to childhood experiences, also considering the role of temperamental features. ST has developed to understand and treat personality disorders [11, 12, 13, 14, 15] and long lasting emotional and interpersonal problems, and it might be useful to heal chronic behavioral problems, such as those related with obesity. The key concepts within the ST approach include Early Maladaptive Schemas (EMSs), Schema Modes and dysfunctional Coping strategies. All of these develop across the life span and are rooted in early childhood and adolescence, where emotional core needs, such as love and nurturance, safety, acceptance, autonomy, limits setting, and so on, might not have been adequately satisfied by caregivers and significant others. Within the ST theoretical framework EMSs are defined as “*extremely stable and enduring themes, comprised of memories, emotions, cognitions, and bodily sensations regarding oneself and one's relationship with others, that develop during childhood and are elaborated on throughout the individual's lifetime, and that are dysfunctional to a significant degree*” [11]. They are considered to have developed through the interaction between child temperament and early experiences of deprivation and/or frustration. For a detailed description of the most relevant schemas within the model, see Table 1. To deal with distress arising from core needs' frustration and schema activation, individuals actuate specific survival strategies, known as coping strategies. These include traditional fight, flight and freeze responses, referring, within the ST language, respectively, to overcompensation (i.e., attacking others, seeking for approval, etc.), avoidance (i.e., using strategies to avoid contact with needs and emotions, dissociation, behavioral avoidance, etc.) and surrender (i.e., submission toward abusive or neglecting relationships) strategies. According to some authors [16] overeating stands for a dysfunctional way to deal with upsetting events, life stressors and overall negative emotions. Waller's schema-based model of eating disorders [17] suggests that the schema process is thought to be central in the purging/bingeing type of eating disorders, where over-eating, and eventual compensation behaviors, are activated in order to reduce, or block, the associated affect following the activation of a schema. Indeed, overeating might originate from a limited repertoire of coping behaviors, associated with underlying dysfunctional schemas. Overall, evidence from previous studies support the hypothesis that overweight and obesity are linked with more dysfunctional schemas, as compared with normal-weight individuals, thus resulting in a more dysfunctional behavior associated with food intake. Another key concept developed within the ST context, refers to Schema Modes, commonly known as modes. These represent the activation in a precise moment, in the here and now, of a specific maladaptive schema or pattern of maladaptive schemas or coping strategy (see Table 2 for a detailed list of modes and their characteristics). As a consequence of EMSs, individuals with personality disorders or other long-standing problems might display dysfunctional modes, in response to specific triggers. To better clarifying the difference between EMSs and modes, some authors [18] refer to the latter as traits and to modes as states. In the case of overweight and obesity one might hypothizes that dysfunctional eating attitudes might represent the activation of a specific coping in response to a stressor or any negative emotional state related to schema activation. Up to now, modes have not been investigated within obese and overweight population.

**Table 1:** Description of the 15 early maladaptive schemas and their domains assessed through the YSQ-SF.

Early maladaptive schemas	Description
Disconnection and rejection Domain	
Emotional deprivation	The belief that others will never met the needs of emotional support
Abandonment	The belief that others will be unavailable or unpredictable in their support and connection
Mistrust/abuse	The belief that others will hurt, take advantage, abuse, and manipulate
Social isolation	A feeling that one is isolated from the rest of the world and other people
Defectiveness/shame	A feeling that one is defective, inferior or invalid
Impaired autonomy and performance Domain	
Failure	The belief that one has failed, or will fail in important life areas of achievement
Dependence	The belief that one cannot afford everyday responsibilities without the help of others
Vulnerability	Fear that inevitable catastrophic events will occur
Enmeshment	Being excessively emotionally involved/connected with important people, at the expense of full individuation or normal social development
Impaired limits Domain	
Entitlement	The belief of being superior to other people, deserving special privileges
Insufficient self-control	Difficulty in self-control and distress tolerance or in restraining excessive emotional expression or impulses
Other directedness Domain	
Subjugation	Always surrendering control to others due to the belief that one is coerced
Self-sacrifice	The belief that one have to meet the needs of other people at the expense of oneself
Over-vigilance and inhibition Domain	
Emotional inhibition	An excessive inhibition of emotions, thoughts, and communications
Unrelenting standards	The belief that one must attain excessively high internalized standards of behavior, usually to avoid criticism

**Table 2:** Description of each mode, including the dysfunctional child, parental and coping modes, and the healthy adult mode, as assessed with the SMI.

**Child Modes**	
Vulnerable Child	Feels lonely, isolated, sad, misunderstood, unsupported, defective, deprived, overwhelmed, incompetent, unloved and unlovable
Angry Child	Feels intense emotion of anger and frustration, the ​core emotional ​(or physical) ​needs ​of the vulnerable child are not met
Impulsive/Undisciplined Child	Acts on ​non-core desires or impulses ​in a selfish or uncontrolled manner, unable to delay short-term gratification; feels intensely angry, enraged, infuriated, frustrated, impatient
Happy Child	Feels loved, connected, satisfied, fulfilled, free, spontaneous
**Coping modes**	
Compliant Surrender	Acts in a passive, approval-seeking, tolerates abuse and/or bad treatment; does not express healthy needs or desires to others
Detached Protector	Cuts off needs and feelings; detaches emotionally from people and rejects their help
Over-compensator	Feels and behaves in a very grandiose, aggressive, dominant, competitive, arrogant, over-controls
**Parent modes**	
Punitive Parent	Feels that oneself or others deserves punishment or blame and often acts on these feelings by being blaming, punishing, or abusive towards self or others
Demanding Parent	Refer to the ​nature ​of the internalized high standards and strict rules
**Healthy adult mode**	
Healthy Adult	Performs appropriate adult functioning, such as working, parenting, taking responsibility, and committing and also practices pleasure in a functional manner

The aim of this study was to investigate maladaptive schemas, modes and dysfunctional coping strategies, and their association with dysfunctional eating behaviors (i.e., bingeing, purging) and other psychological variables, in overweight and obese individuals. A normal weight age and gender matched sample was recruited as control group. Further, we explored the role of coping mechanisms in predicting and mediating between early maladaptive schemas and dysfunctional eating behaviors.

## 2 Materials and methods

### 2.1 Participants

Seventy-five Caucasian volunteers were recruited through an online survey (www.mturk.com). After providing instructions and informed consent, participants fulfilled several self-report measures in one single session. Measures were administered in a random order. All participants reported information about their age, gender, Body Mass Index (BMI), level of formal education, employment and marital status. BMI rates were used to select the normal weight and overweight/obese participants. Additional information about eating related attitudes and other variables were collected, including frequency of binge and/or vomiting/purging compensating behaviors, eventual on-going psychotherapy and psychopharmacological treatment, and relatives' mental illness. The study was conducted according to the Declaration of Helsinki guidelines and it was approved by Guglielmo Marconi University’ Ethical Committee.

### 2.2 Measures

The following measures were administered, with Cronbach's alpha coefficient being calculated for each test.

*The Eating Disorder Inventory 3* (EDI-3; [19]) is a self-report questionnaire widely used to assess symptoms and psychological features of eating disorders. It represents an expansion and improvement of the earlier versions of the EDI. It consists of the same 91 questions with scores ranging from 0 to 4. The EDI-3 also consists of more general psychological trait subscales, investigating low self-esteem, personal alienation, interpersonal insecurity, interpersonal alienation, interoceptive deficits, and emotional dysregulation, affective and interpersonal problems, and overall levels of general psychopathology. An additional score is derived from the combination of three clinical subscales, yielding the eating disorder risk composite score, a measure that provides clinical information about overall eating disorder severity. The reliability of these index scores collected from eating disorder patients appears excellent (Cronbach's α = .90–.97).

*The Centre for Epidemiological Studies - Depression Scale* (CES-D; [20]) is a 20-item self-report measure designed to measure depressive symptoms during the past week, in the general population. Total score ranges from 0 to 60. Standard cut-offs are >16 for mild depression and >23 for clinical depression. Cronbach's alpha reliability coefficient was α = .80, which implies acceptable internal consistency.

*The Young Schema Questionnaire - Short form* (YSQ-SF, [21]) is a 75-items questionnaire assessing 15 EMSs. Each scale consists of five items, and participants are asked to rate the items using a 6-point Likert scale (from 1 = completely untrue of me, to 6 = describes me perfectly). The 15 schemas included in the test are: Abandonment, Mistrust/Abuse, Emotional Deprivation, Defectiveness/shame, Social Isolation, Dependence, Vulnerability to Harm or Illness, Enmeshment/undeveloped self, Failure, Entitlement, Insufficient Self-Control, Subjugation, Self-Sacrifice, Emotional inhibition and Unrelenting Standards. The YSQ-SF showed a high internal consistency, with a Cronbach's alpha coefficient (α) of .97.

*The Young - Rygh Avoidance Inventory* (YRAI, [22]) contains 40 items that assess schema avoidance. Each item is rated on a 6 point Likert scale from 1 (“completely untrue of me”) to 6 (“describes me perfectly”). Usually, for research purposes, Young and other therapists divided YRAI items into 14 subscales, based on what they believe to be different avoidant strategies or symptoms, however, according to our aims and considering the weak reliability of this test, we extracted only three types of scores within the questionnaire, namely: 1) intra-psychic (i.e., denial of memories, excessive rationality and control, etc.), 2) behavioral (i.e., substance abuse, distraction through activity, avoidance of upsetting situations, self-soothing behaviors etc.), and 3) dissociative (i.e., passive blocking of upsetting emotions, passive distraction through fantasy, day-dreaming or television) avoidance coping strategies. The internal consistency of the YRAI was quite acceptable, with α = .84.

*The Young Compensation Inventory* (YCI, [23]) contains 48 items assessing strategies used for schema compensation. Each item is rated on a 6 point Likert scale from 1 (“completely untrue of me”) to 6 (“describes me perfectly”). Young observed that the same form of overcompensation could be used to cope with different schemas. The YCI lists some of the most common schemas being associated with each of the items on the test. Higher scores at YCI are indicative of greater employ of compensation strategies. These high scores indicate that patient overcompensates for emotions connected with his or her schemas. Cronbach's alpha reliability coefficient was very good, with α = .93.

*The Schema Mode Inventory* (SMI, [24]) is a 124 self-descriptive statements that covers 14 modes (i.e., Vulnerable child, Angry child, Enraged child, Impulsive child, Undisciplined child, Happy child, Compliant child, Detached protector, Detached self-soother, Self-aggrandizer/Bully and attack mode, Punishing parent, Demanding parent, Healthy adult), where subjects have to rate the frequency on a 6-point scale (ranging from “never or hardly ever” to “always”). The higher the score, the more frequent were the manifestations of the modes. Items of the SMI reflected emotions, cognitions or behaviors. Internal consistency coefficient was α = .96, showing an excellent reliability.

### 2.3 Analysis

Data were analyzed using SPSS version 20.0 software (SPSS Inc., Chicago, IL). In a first analysis we explored descriptive characteristics and performed group comparison analyses in order to explore between groups’ differences. Correlation analyses were performed within the overweight/obese group in order to detect associations between BMI, dysfunctional eating patterns, and general level of psychopathology and schemas, coping styles and modes. Further, stepwise multiple regressions were performed to identify which variables among all schema-related constructs accounted for degrees of obesity, bingeing and bulimic symptoms. In a last analysis, mediation models were estimated with maladaptive schemas (assessed through the YSQ) and parental modes (as measured with the SMI) as predictor variables, dysfunctional eating behaviors as dependent variables and coping strategies considered as possible mediating factors.

## 3 Results

### 3.1 Descriptive statistics

An overweight/obese (BMI = 32.80, range 25.0–55) and normal-weight group (BMI = 21.10, range 17.5–24.9) was obtained. Demographic data within the two groups are reported in Table 3. Groups did not differ in gender distribution, or in their level of formal education, marital status, and levels of depression. Conversely, overweight and obese subjects reported significantly higher scores in dysfunctional eating attitudes (i.e., binge and vomit frequency) and many EDI subscales (i.e., bulimia, risk of developing an ED, body dissatisfaction, etc.). Within the overweight/obese group, 49% participants reported bingeing episodes occurring on a monthly or daily basis, whereas the remaining 61% reported no binging episodes. See Table 3 for details and statistical significance.

**Table 3:** Descriptive and group analyses on demographic, dysfunctional eating attitudes and psychological variables. Independent T-tests and Chi-Squares were performed to calculate, respectively, parametric and non-parametric variables between groups’ (see last column for levels of significance). Abbreviations: BMI = Body Mass Index; CES-D = Centre for Epidemiological Studies - Depression Scale; EDI-3 = Eating Disorder Inventory*-*3*;* SD = Standard Deviation; ns = not significant difference.

	Normal weight sample N = 37	Overweight/Obese sample N = 48	*P* value
BMI [SD]	21.1[2.3]	32.8[8.0]	0.00
Mean age [SD] years	35.1[13.7]	38.8[13.5]	ns
Gender %	45.9% female	54.1% female	ns
Level of formal education %	19% bachelor 27% college 24% master degree	35% college 31% bachelor 10% master degree	ns
Marital status %	45% single 32% married	43% single 33% married	ns
CES-d total Mean score [SD]	18.97[8.4]	21.19[9.1]	ns
Binge frequency (1= never, 2=monthly, 3=weekly, 4= daily) [SD]	1.3[0.7]	2[1.5]	0.03
Vomit episodes frequency (per month) [SD]	0[0]	2[0.9]	0.02
Bulimia (EDI-3) [SD]	3.7[6.8]	11.1[9.9]	0.00
Body dissatisfaction (EDI-3) [SD]	10.1[11.8]	21.3[13.3]	0.00
Low self-esteem (EDI-3) [SD]	4.8[6.3]	9.4[6.8]	0.02
Emotional dysregulation (EDI-3) [SD]	9.8[4.5]	12.8[5.8]	0.01
Inadequacy (EDI-3) [SD]	12.2[10.7]	19.5[11.8]	0.04
Affective problems (EDI-3) [SD]	15.7[12.2]	22.8[13.8]	0.01
Risk of developing an ED (EDI-3) [SD]	20.9[24.4]	44.8[28.0]	0.00

### 3.2 Between groups’ comparisons

**Fig. 1:**
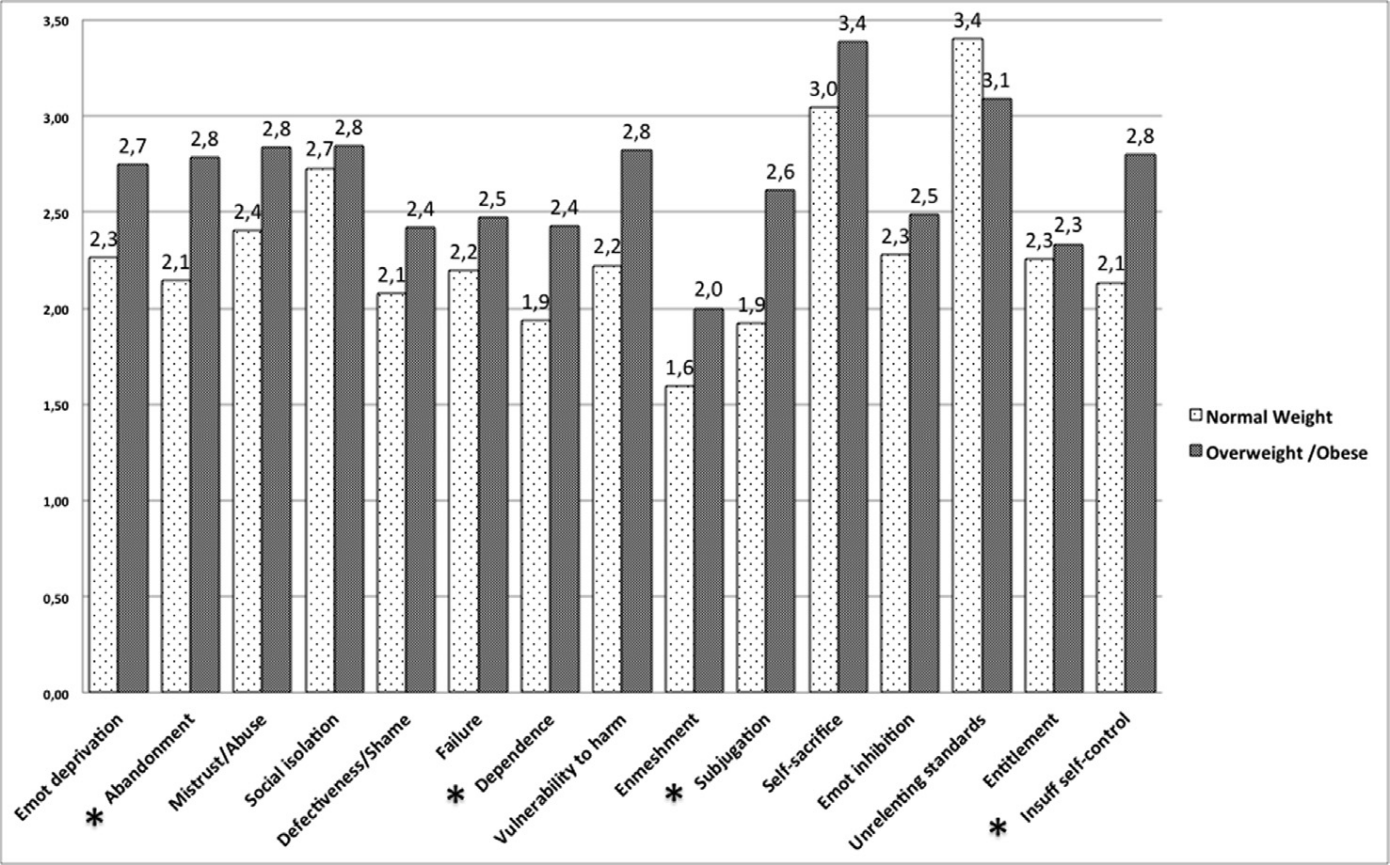
Mean scores and significant differences between the two groups in the Early Maladaptive Schemas (YSQ). Independent t-tests were performed. Statistical significant significances are reported *p < 0.05.

**Fig. 2:**
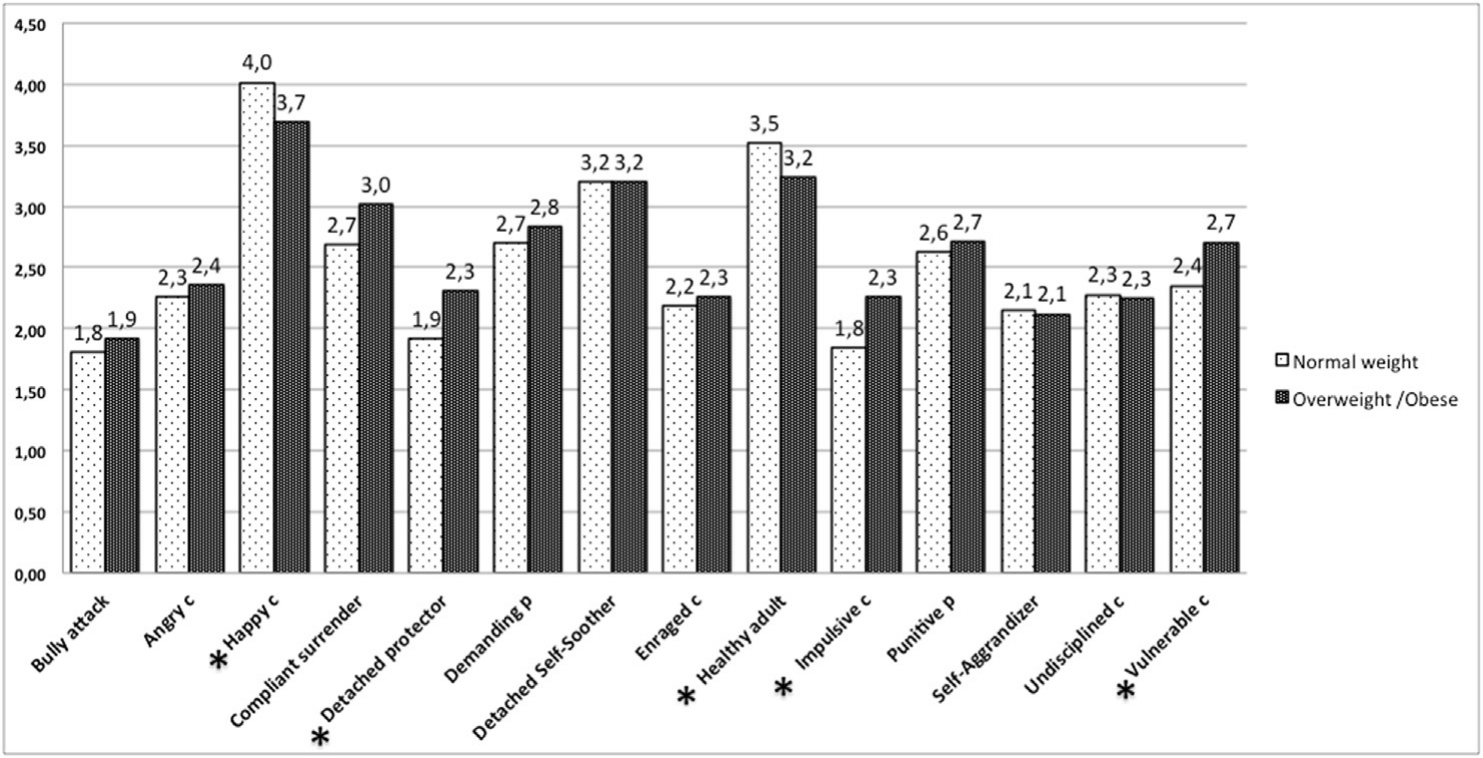
Mean scores and significant differences between the two groups in the Schema Modes (SMI). Independent t-tests were performed. Statistical significant significances are reported *p < 0.05. Abbreviations: c = child mode, p = punitive parent mode.

In order to compare the two groups, t-independent sample tests were performed to detect for differences in schemas, modes and avoidant and overcompensating coping strategies. Overweight and obese subjects reported significantly more pervasive Abandonment (t(83) = -2.22, p < .05), Dependency (t(83) = -2.82, p < .005), Subjugation (t(83) = -2.54, p < .01), and Insufficient self-control schemas (t(83) = -2.91, p < .005; see Fig. 1 for mean scores). Accordingly, among modes, the Vulnerable and Impulsive child and the Detached Protector coping mode (t(83) = -2.19, p < .05), were higher, whereas the Happy child (t(83) = 2.55, p < .05), and the Healthy adult (t(83) = 3.45, p < .001), were stronger in the normal weight participants (see Fig. 2 for mean scores). No between groups’ differences were detected in the YCI, while within the YRAI only the behavioral avoidant strategies subscale (t(83) = -2.01, p < .05), was more pervasive in the overweight/obese group (M = 2.68, SD = 0.95), than in the normal weight group.

### 3.3 Correlation analyses

Within the overweight/obese group significant associations were detected. Overall, binge frequency was positively associated with all maladaptive schemas and dysfunctional modes. BMI index correlated with the insufficient self-control schema. Positive correlations between schemas, modes and copying styles were also observed in relation to bingeing, bulimic symptoms’ severity, vomit frequency and an overall risk of developing an eating disorder. See Tables 4, 5 and 6, for details.

**Table 4:** Correlations between early maladaptive schemas assessed with the YSQ, eating related variables and psychological variables associated with eating disorders (EDI3 subscales). Abbreviations: BMI = Body mass index, ED = eating disorder.

	Emotional Deprivation	Abandonment	Mistrust/Abuse	Social isolation	Defectiveness/Shame	Failure	Dependency	Enmeshment	Subjugation	Self-sacrifice	Emotional Inhibition	Unrelenting Standards	Entitlement/grandiosity	Insufficient self-control
BMI	-	-	-	-	-	-	-	-	-	-	-	-	-	.44
Binge	.44	.66	.61	.36	.62	.70	.61	.72	.63	.31	.61	.42	.61	.70
Bulimia	-	.48	.49	-	.33	.50	.35	.55	.52	-	.40	.34	.38	.52
Vomit	-	-	-	.30	.41	.31	.34	-	-	-	-	-	.30	-
Risk of an ED	.32	.54	.39	-	-	.36	-	.35	.46	-	.29	.33	-	.55

**Table 5:** Correlations between schema modes assessed with the SMI, eating related variables and psychological variables associated with eating disorders (EDI3 subscales). Abbreviations: BMI = Body mass index, CM = coping mode; ED = eating disorder.

	Bully/attack CM	Angry Child	Happy Child	Compliant/surrender CM	Detached Protector CM	Demanding Parent	Detach Self-Soother CM	Enrage Child	Healthy Adult	Impulsive Child	Punitive Parent	Self-aggran-dizer CM	Undisciplined Child	Vulnerable Child
BMI	-	-	-	-	-	-	-	-	-	-	-	-	-	-
Binge	.47	.56	-.35	.48	.59	.58	-	.51	-	.66	.61	.58	.62	.58
Bulimia	.35	.42	-.29	.41	.39	.52	-	.51	-	.51	.51	.42	.42	.35
Vomit	.32	.29	-.23	-	.30	.29	.29	.25	-	.34	.37	.24	-	.28
Risk of an ED	-	.23	-.41	.41	.26	.37	-	-	-.22	.28	.31	-	.22	.46

**Table 6:** Correlations between avoidance coping strategies assessed with the YRAI, eating related variables and psychological variables associated with eating disorders (EDI3 subscales and CES-d). Abbreviations: BMI = Body mass index, ED = eating disorder.

	Intra-psychic Avoidance	Behavioral Avoidance	Body dissatisfaction	Low self-esteem	Emotion dysregulation	Affective problems	Depression
BMI	-	-	.41	-	-	-	-
Binge	.72	.71	.29	.42	.52	.56	.54
Bulimia	.46	.56	.55	.51	.76	.76	.40
Vomit	.39	-	.41	.32	.33	.37	.25
Risk of an ED	.43	.49	.88	.37	.52	.54	.43

### 3.4 Multiple regression analyses

Schema therapy constructs (i.e., schemas and modes) were regressed on BMI and dysfunctional eating behaviors (i.e., bulimic symptoms, bingeing and vomit frequency). We considered as an acceptable value for coefficient of determination R^2^ > 0.5. Results showed that frequency of bingeing was predicted by the Abandonment, Enmeshment/undeveloped self and Failure schemas (F(3,44) = 38.84, p < .000, R^2^ = .70), by the Impulsive and Undisciplined child and the Punitive parent modes (F(2,45) = 31.38, p < .000, R^2^ = .58), and by the intra-psychic avoidance coping (F(1,46) = 53.37, p < .000, R^2^ = .53).

### 3.5 Mediation models

Our mediation hypotheses were tested using the SPSS macro (Hayes's PROCESS), calculating a path analysis to test if dysfunctional coping strategies mediated between maladaptive schemas and dysfunctional eating behaviors/BMI index. In the first step the presence of a significant effect that may be mediated between the predictor variable X and the outcome variable Y (path c, also called “total effect”) is established. Step 2 calculates whether the predictor variable X affects the mediator variable (path a). Step 3 establishes that the mediator variable affects the outcome variable Y (path b). Finally, the 4^th^ step establishes if the mediator variable fully mediates the X–Y relationship (path c', also called “direct effect”); if so, the effect of X on Y controlling for the mediator should be zero.

**Fig. 3:**
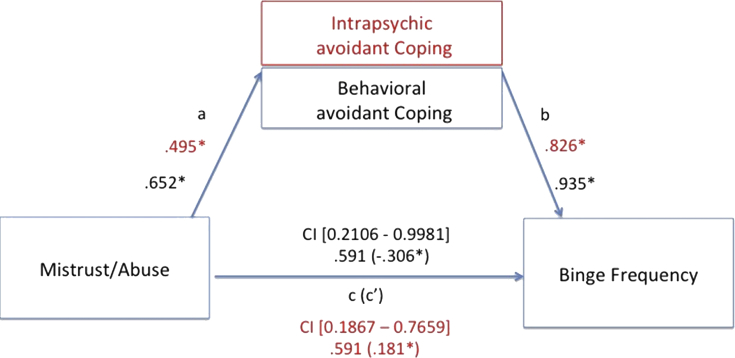
Direct and mediated pathways for the relationship between the mistrust/abuse Early Maladaptive Schema and binge frequency, as mediated by both intrapsychic (in red) and behavioral (in black) avoidant coping strategies. Note: *p < .05.

**Fig. 4:**
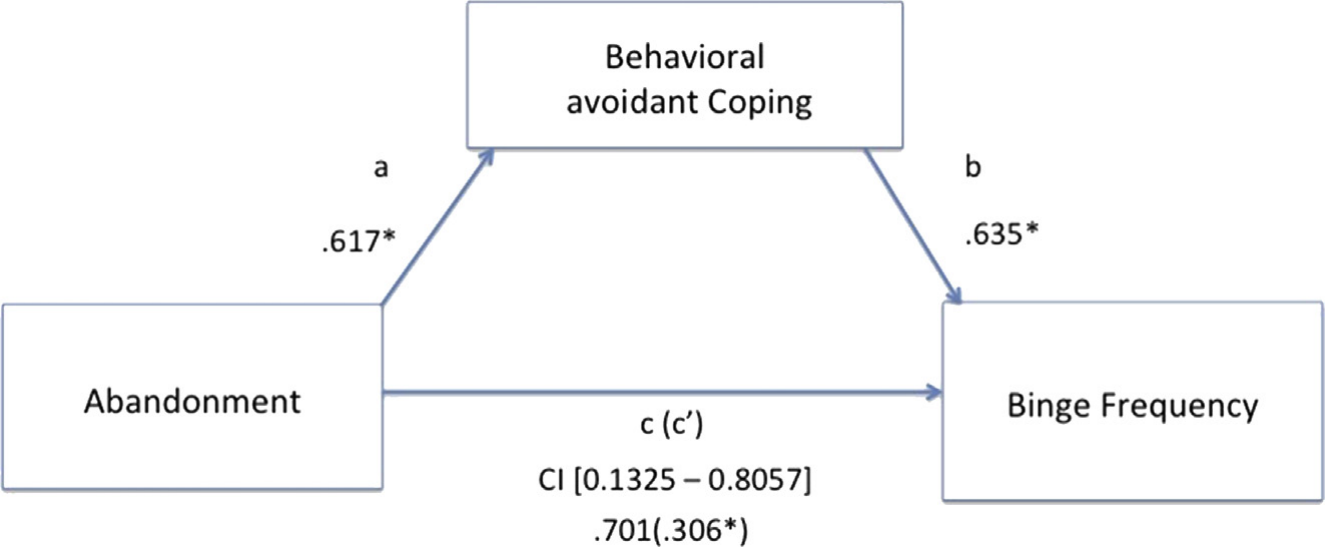
Direct and mediated pathways for the relationship between the abandonment Early Maladaptive Schema and binge frequency, as mediated by behavioral avoidant coping strategies. Note: *p < .05.

**Fig. 5:**
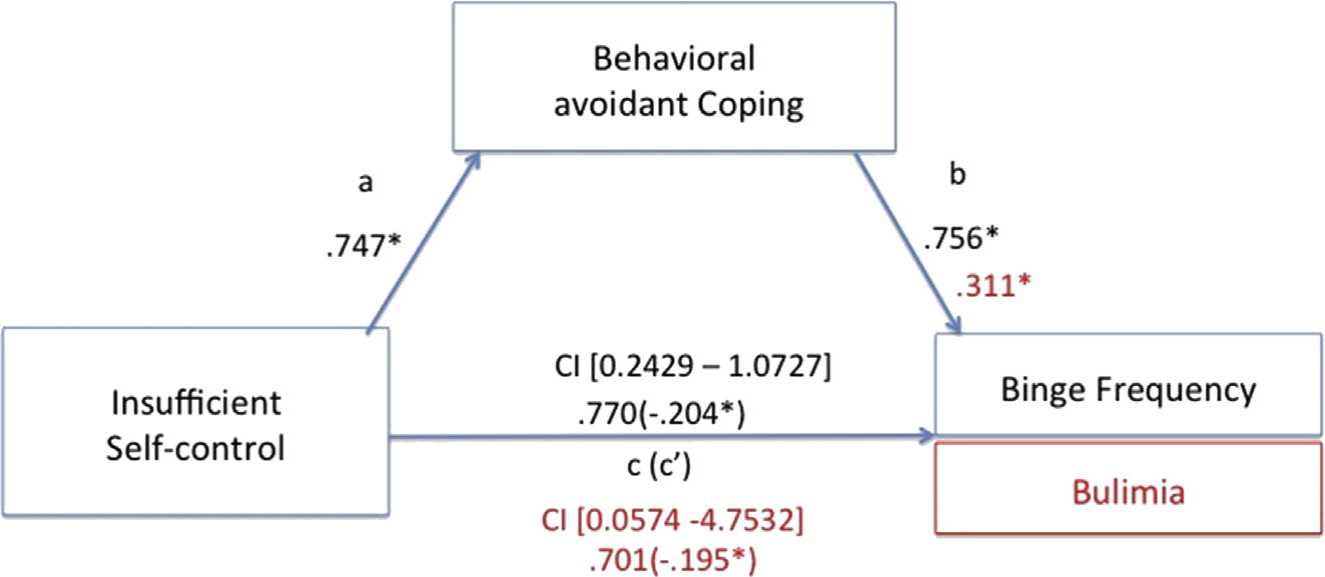
Direct and mediated pathways for the relationship between the insufficient self-control Early Maladaptive Schema and binge frequency (in black) and bulimic symptoms' severity (in red), as mediated by behavioral avoidant coping strategies. Note: *p < .05.

**Fig. 6:**
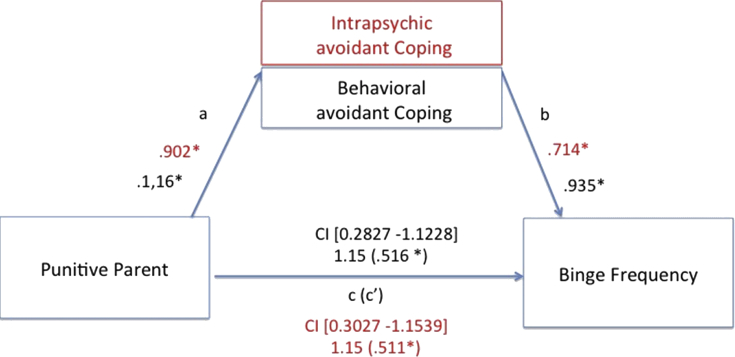
Direct and mediated pathways for the relationship between the punitive parent mode and binge frequency, as mediated by both intrapsychic (in red) and behavioral (in black) avoidant coping strategies. Note: *p < .05.

We tested for specific mediation models, selecting those EMSs that resulted being significantly higher than the control group. Additionally, due to the well-known role of abuse in obesity [24], also the mistrust/abuse schema was added as possible predictor variable, together with the demanding and punitive parental modes. All avoidant coping strategies were introduced as mediators, while BMI, binge frequency and bulimic symptoms' severity were set into the model as dependent variables. Among all mediation models, the only significant ones included the abandonment, mistrust/abuse and insufficient self-control schemas and the punitive parental mode as significant predictors of bingeing frequency and bulimic symptoms. The abandonment, mistrust/abuse and insufficient self-control schemas explained binge frequency through behavioral and intra-psychic coping strategies. Further, both coping strategies also mediated between the punitive parent mode and bingeing severity. Finally, bulimic symptoms’ severity was explained by the insufficient self-control schema, mediated by behavioral avoidant coping. No significant models emerged with BMI rates as explanatory variable. See from Figs. 3, 4, 5, and 6, for details.

## 4 Discussion & Conclusions

This study investigated early maladaptive schemas, modes and coping strategies in overweight/obese individuals, in comparison to normal weight controls. Also we explored the association, and possible mediating effect, of schema related variables and dysfunctional eating patterns, within the overweight/obese group.

Previous studies [15, 26, 27, 28, 29, 30, 31] have shown an association between early maladaptive schemas (i.e., abandonment, insufficient self-control social isolation, failure to achieve, defectiveness/ shame, dependence/ incompetence, entitlement/ grandiosity) and overeating, and other eating-related dysfunctional attitudes, such as food addiction and bingeing. Similarly, in our study overweight/obese participants reported more intense abandonment, dependence, subjugation and insufficient-self control schemas, compared with normal weight subjects. We suggest that the abandonment schema, which results from the frustration of primary core needs such as care and nurturance by the caregiver, might be the first one to develop, with insufficient-self control developing later on in life, as arising from the frustration of later emotional core needs. The insufficient-self control schema originates from the absence of any parental limit or rule and prevents the ability to tolerate negative emotions, impulses and frustrations in general. We found that this schema was the only one to be significantly associated with BMI rates, and it might be the forerunner for future avoidant coping mechanisms. Coherently, the insufficient-self control and abandonment schemas reflect the impulsive and vulnerable child modes, respectively, who were found to be significantly more pervasive in the obese group (vs healthy control). Moreover, mediation models showed that both schemas predicted binge frequency, being fully mediated by behavioral avoidant coping strategies (i.e., self-soothing behaviors, distraction through activity, avoidance of upsetting situations, etc.) that might be represented by the detached protector coping mode. This mode was also found to be particularly elevated in the obese group, being positively associated with frequency of bingeing episodes, bulimic symptoms' severity, and with an overall risk to develop an eating disorder. Taken together, these data suggest the possibility that obese individuals might have grown-up in a neglecting, or an over-protective, environment where they did not have the possibility to mature a functional way to deal with negative feelings and to tolerate frustration. Instead, they might have developed some self-soothing detaching behaviors, mainly referring to food intake and, eventually, later on, to some food-related compensating behaviors (i.e., purging). Although the surrender coping mode and the impulsive child mode might overlap, the ST model states there are some differences between the two. The impulsive child mode is a natural state of a child, uninhibited and free, and might be associated with an insufficient self-control schema. When the ‘Impulsive Child’ mode is activated the individual shows low frustration tolerance and cannot delay short-term gratification for the sake of long-term goals, appearing spoiled, lazy, out of control [11]. On the other hand, the surrender coping mode is more related to the behavioral manifestation of the insufficient self-control schema, where the individual does not activate any effective strategy to cope with frustration and overall negative emotions.

Although we did not find a significant difference between groups in the mistrust/abuse schema, there is great evidence highlighting the role of abuse, especially sexual mishandling, in the childhood of obese individuals (see [25], for a review). In one study, childhood sexual abuse was found to be the strongest predictor of later bingeing [32], while other studies revealed that obese with Binge Eating Disorder (BED) reported more abusive experiences, than non-BED obese [33, 34]. In fact, our correlation analyses showed that the mistrust/abuse schema was positively associated with binge frequency, bulimic symptoms’ severity, and with an overall risk of developing an eating disorder. Finally, being mediated by intra-psychic and behavioral avoidance coping strategies, the mistrust/abuse schema was a significant predictor of binge frequency. Again, these data confirm the usefulness of avoidant coping strategies in dealing with distressing emotions, suggesting the need for future studies deepening the role of the mistrust/abuse schema in obese with and without BED.

Compared against normal weight participants, obese subjects reported higher dependence and subjugation schemas. These EMSs refer, respectively, to the belief that one cannot afford everyday responsibilities without an external help, and that own desires and needs must be surrendered to those of others. The roots of both schemas are related to the frustration of core needs related to autonomy and self-efficacy, sense of identity and feelings of competence. In fact, already in 1973, Hilde Bruch [35] stated the extremely dependent and enmeshed relations obese patients reported having with their relatives. As a consequence, both obese children and adults were unable to appropriately identify their own needs, to regulate their impulses and to develop and maintain a stable sense of identity, autonomy and competency. In such scenario, food might represent a way to surrender to parental enmeshment, and to a lack in self-confidence [36]. Additionally, it absolves a rewarding function as related to self-soothing and needs and wishes avoidance.

Finally, an interesting finding refers to the role of the punitive parent mode. This mode usually reflects parental internalized voices, being characterized by punishing or blaming messages towards the self or others. Typically, this mode refers to the punitive early maladaptive schema, which, unfortunately, is not assessed through the YSQ-SF, used in the study. Our findings showed that both intra-psychic and behavioral avoidance coping strategies fully mediate the association between punitive parent mode and binge frequency. One possible explanation might consider the punitive parent mode as the introjection of parental feeding practices, where parents were used to reward their children with food for their appropriate behaviors, while punishing them by taking away food for mistakes or wrong behaviors. This educational behavior is known as *instrumental feeding* and has been related with emotional eating, tendency to overeat [37], snacking [38, 39], and bulimic symptomology [40]. Other studies showed that college students who had reported instrumental feeding practices in their childhood, displayed a higher BMI and engaged in greater binge eating in adulthood, compared to individuals who did not report any of these experiences [41]. Finally, Mason et al. [42] observed that overweight and obese individuals reporting parental instrumental feeding practices were more at risk for engaging in binge eating, particularly in response to negative affect. This means that, when experiencing distressing emotions, obese individuals may turn to high calories, high palatable foods and bingeing behaviors to cope with their negative emotional states (as suggested by Waller, [17]). As well, another possibility might refer to binge eating as an expression of ‘acting out’ the internalization of punitive impulses into aggressive self-directed binge eating episodes.

**Fig. 7:**
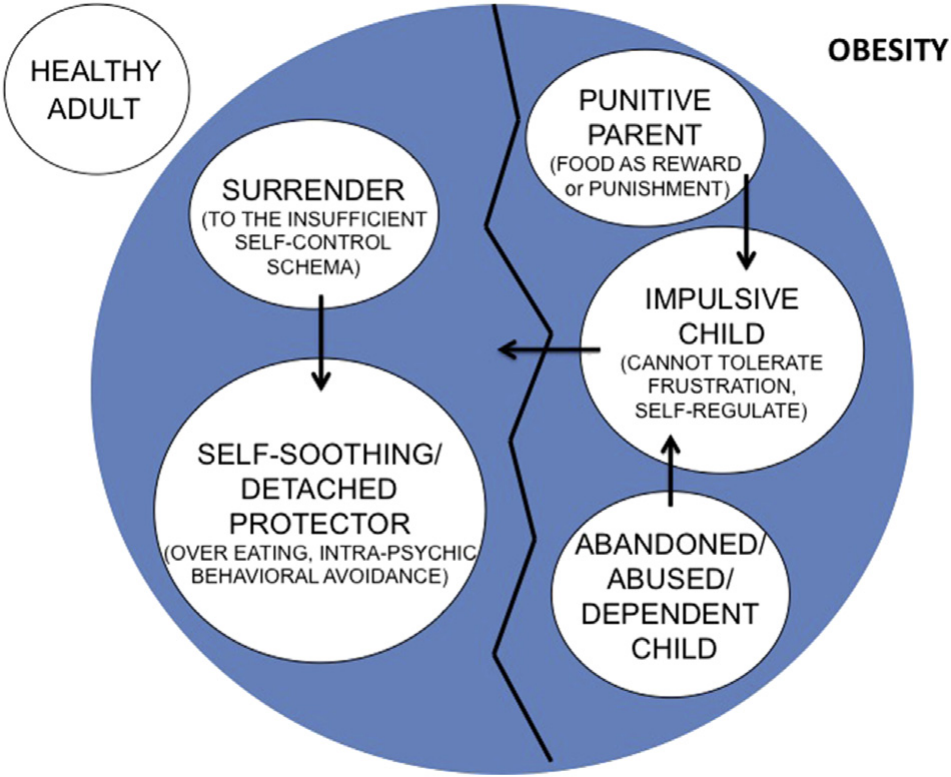
Proposed schema mode model of Obesity (as adapted from Arntz 2012), adapted to Max's formulation.

Overall, our findings show how current dysfunctional eating behaviors associated with bingeing and bulimic symptoms in overweight and obese individuals might be rooted in long-lasting abnormal emotional and behavioral attitudes toward food. The Schema Therapy model traditionally focuses on early maladaptive schemas, and their related current modes, and helps to eradicate and reduce their associated dysfunctional coping strategies, in order to allow the client to access early negative experiences through emotion-focused techniques (i.e., imagery with rescripting). Fig. 7 illustrates how the present findings might be exemplified in a schema mode model of obesity, as adapted from Arntz [13] model of personality disorders. This graphical representation might also illustrate the case conceptualization of Max, a 40 years-old client seeking for help because of his extreme overweight (BMI = 33.6), which caused him a lot of discomfort in almost all domains of his life. Max was a wealthy man. His father used to be very aggressive and punitive towards him in his childhood, but also in adulthood. Max's mother was over-protective and anxious towards her son, and very submissive towards her husband who used to yell at her very often. At the age of 10, an older mate had sexually abused Max, and he had never disclosed to anyone about this fact. In those early years the child started eating a lot, using food both as a self-soothing coping strategy to deal with his emotional distress, and as a surrendering coping towards his insufficient self-control schema (the surrender coping mode). Additionally, weight gain was a way to become more unappealing, in order to discourage any sexual consideration by others. Max's current functioning can be illustrated in Fig. 7, where the intra-psychic (i.e., denial) and behavioral (i.e., over-eating/bingeing) avoidant coping strategies – called the Self-soother/Detached Protector coping mode - derive from the attempt to reduce the tension between Max's Punitive Parent mode harsh messages, and his emotional needs (i.e., attachment, protection, competence and autonomy, related to the Abandoned, Abused and Dependent child modes) and inability to tolerate frustrations and control impulses (Impulsive child mode). These represent, respectively, the avoidant coping modes, the parental mode and the vulnerable child modes. Finally, in the left corner of the figure the Healthy Adult mode is shown. Identifying each of these modes and their functions is the first steps of ST intervention. Further on, the main goals of ST include: 1) addressing and satisfying the vulnerable child's core emotional needs, in a safe therapeutic relationship, 2) de-potentiate the punitive parental mode, 3) reduce dysfunctional coping mechanisms and, transversally across treatment, expanding the healthy adult mode in order to help the client dealing with the child's needs and emotions in a healthier way.

The findings of the present study highlight the role of early schema dysfunction, related schema modes, and abnormal eating behavior in overweight and obesity. Some limitations of the study include the small sample size, the use of self-report questionnaires, online recruitment and the lack of discrimination between over-eaters with and without Binge Eating Disorder (BED) symptoms, within the experimental group. Although our subjects were recruited from a community sample, allowing generalization to overweight/obese individuals, future studies should include patients from a treatment setting. Prospective studies are also required to further clarify the nature of the association between ST related constructs (i.e., schemas, modes and coping strategies) and abnormal eating behavior in obesity, so that clearer statements can be made regarding the putative causal and maintenance role of schemas in triggering eating problems and, ultimately, weight increase.

## Declarations

### Author contribution statement

B. Basile: Conceived and designed the experiments; Performed the experiments; Analyzed and interpreted the data; Contributed reagents, materials, analysis tools or data; Wrote the paper.

K. Tenore: Conceived and designed the experiments; Contributed reagents, materials, analysis tools or data. F. Mancini: Analyzed and interpreted the data; Wrote the paper.

### Funding statement

This research did not receive any specific grant from funding agencies in the public, commercial, or not-for-profit sectors.

### Competing interest statement

The authors declare no conflict of interest.

### Additional information

No additional information is available for this paper.
